# Prevalence of teenage pregnancy and the associated contextual correlates in Rwanda

**DOI:** 10.1016/j.heliyon.2020.e05037

**Published:** 2020-10-08

**Authors:** Dieudonne Uwizeye, Ruben Muhayiteto, Evelyne Kantarama, Simeon Wiehler, Yusuf Murangwa

**Affiliations:** a School of Governance, University of Rwanda, Kigali, Rwanda; b National Institute of Statistics of Rwanda, Kigali, Rwanda; c School of Medicine and Pharmacy, University of Rwanda, Kigali, Rwanda; d African Population and Health Research Center, Nairobi, Kenya

**Keywords:** Sociology, Psychology, Quality of life, Social organisation, Human rights, Teenage pregnancy, Parents' empowerment, Household characteristics, DHS, Rwanda

## Abstract

The rate of teenage pregnancy remains unacceptably high in most developing countries. In Rwanda, studies show a rapid increase over the past two decades despite the political achievements of women's empowerment, and efforts to curtail child sexual abuse. Unfortunately, the current knowledge of the household determinants of teenage pregnancies in Rwanda is limited, as recent studies have focused on providing numbers with little analysis of proximate causal factors or focused on the individual determinants. The study uses secondary data from the recent Rwanda Demographic and Health Survey (RDHS: 2014–2015) to analyse household factors associated with teenage pregnancies in Rwanda. In addition to descriptive analysis, we ran logistic regression models to determine the level of association between teenage pregnancy and household socioeconomic characteristics.

Results indicate that marital status and age of household head, household size, number of bedrooms given the size of the household, and the educational level of the household-head are significantly associated with teenage pregnancy (p < 0.01). Teen girls from small households are more likely to get pregnant than those from large families, while financial, social and educational empowerment of parents, and harmonious household contexts contribute to lessening the cases of teenage pregnancy. It indicates that social and economic support to teen girls which include parental supervision, guidance, and financial care are essential aspects to consider in order to reduce teenage pregnancy rates. The study suggests that in addition to efforts directed to teens themselves, strategies for reducing teen pregnancies should focus on a range of household-level contexts that form two broad categories: empowering parents and maintaining parents' harmonious decisions on teen girls.

## 1 Introduction

Despite the decline of global adolescent birth rates from 65 to 47 births per 1000 women between 1990 and 2015, teenage pregnancies remain unacceptably high especially in sub-Saharan Africa (Odejimi et al., 2016; Odimegwu and Mkwananzi, 2016), Latin America and the Caribbean (Treviño-Montemayor, 2018). Several empirical studies have investigated factors linked to teenage pregnancies in different countries, finding that socioeconomic status of teens' households were significant correlates. Lower socioeconomic status was associated with adolescent pregnancy in Tanzania (Nyakubega, 2008), Malawi (Kost et al., 2017) and in Nigeria, where adolescent girls whose parents were poor were likely to engage in premarital sex which eventually leads to childbearing (Ajala, 2014; Odimegwu and Mkwananzi, 2016). Adolescent girls from households that live in poverty were involved in sexual relationships in exchange for gifts such as money, clothes, and other goods (Nwogwugwu, 2013; UNFPA, 2013).

Further, accumulating evidence indicates that reduced access to information on contraceptives and barriers to reproductive health services among adolescents and young adults were associated with teenage pregnancy (Kaphagawani and Kalipeni, 2017; Treviño-Montemayor, 2018). For instance, in Tanzania, young girls with incomplete information on reproductive health were more likely to become pregnant before the age of 19 (Nyakubega, 2008) while in Kenya Were (2007) found that lack of access to education opportunities, sex education and information regarding contraceptives predisposed teen girls to pregnancies.

Indeed, widespread teenage pregnancies in low-income countries pose serious health challenges and restrictions to socioeconomic opportunities not only for the young girls but also for their children and families. Since teenagers are still at an early developmental stage of life, being a mother at this age makes it challenging to take the appropriate economic, social, and psychological responsibilities. In most cases, by the time a young girl becomes pregnant, especially in a low-income countries context, she loses the opportunity to education and thus exposes herself to limited economic prospects. Once pregnant, teenage girls, in most of the developing countries, are more likely to drop out of school (Ajala, 2014; Were, 2007) hindering their chances for better-paying jobs. Marisa and Marisa (2018) discussed that in Zimbabwe girls who avoid pregnancy are more likely to stay in school and eventually secure a more lucrative job or other income-earning opportunities while teenage mothers get trapped in poverty and often become an economic burden to their family and country.

Furthermore, adolescent pregnancy in low income countries context remains a significant contributor to maternal and child mortality (Habitu et al., 2018) and to unsafe abortion. The available data on abortion among 15–19 indicates that 3.9 million unsafe abortions occur globally each year, leading to increased danger of chronic health problems among the teen mothers (UNFPA, 2013). In the context of Rwanda, for instance, babies of teen mothers are 50% more likely to be stillborn, die early, or develop acute and long-term health problems (National Institute of Statistics of Rwanda_NISR, 2015). Also, a study conducted in Rwanda has shown that many teens, upon realizing they are pregnant, attempt unsafe abortions, and some have ended in serious genital organ damage or in death (Basinga et al., 2012).

Abortion in Rwanda is generally illegal. It is only permissible in cases the pregnancy is associated to health risks, the pregnant person is a child (under the age of 18), the pregnancy is a result of rape, forced marriage or incest (Government of Rwanda, 2019). In addition to social, mental and clinical risks associated to abortion, teenage pregnancy alone remain a serious family concern as it affects family cohesion and induces stigma (Mollborn, 2017) and, in Rwanda, health care providers have been reluctant to safe abortion care even within the legal framework (Påfs et al., 2020).

The increase in teenage pregnancy rates in Rwanda in recent years is worrisome. The data from NISR (Table 1) indicate that in 2007/2008 to 2014/2015 teenage pregnancy increased from 5.7% to 7.2% of the teen girls countrywide, and from 14% to nearly 21% among young girls aged 19 (NISR, 2009; 2012; 2015).

**Table 1:** Prevalence and trend of teenage pregnancy in Rwanda.

Characteristics of teen girls	2007		2010		2014/2015	
	Percent pregnant	Number of women	Percent pregnant	Number of women	Percent pregnant	Number of women
**Age**						
15	0.0	265	0.0	677	1.0	676
16	2.6	274	0.8	655	2.0	559
17	2.4	267	3.3	530	4.3	518
18	8.6	293	9.7	605	11.5	557
19	14.1	288	20.3	478	20.8	469

**Family Residence**						
Urban	5.3	275	5.4	447	7.9	575
Rural	5.8	1,112	6.2	2,499	7.1	2,204

**Province**						
City of Kigali	9.1	155	6.6	332	10.2	359
South	5.6	371	4.9	642	5.6	668
West	5.2	320	5.4	762	5.8	597
North	6.0	234	5.7	503	4.9	526
East	4.5	308	7.9	707	10.7	629

**Education level**						
No education	5.1	104	24.9	87	12.7	41
Primary	6.3	1,076	6.1	2,132	9.2	1,632
Secondary and higher	2.9	207	3.6	727	4.3	1,106

Source: (NISR, 2009; 2012; 2015).

According to the data (Table 1), teenage pregnancy was consistently higher in rural areas than in urban in the 2007 and 2010 surveys. However, in 2015, the urban areas came first with 8% of the teens living in town, against 7% presented in rural areas. Kigali city and the Eastern province present the highest increase of teenage pregnancy in recent years from nearly 7% to 10% and from 8% to nearly 11% in 2010 and 2015 survey respectively.

The increase comes despite the government interventions through improved sex education and teenage mentoring mainly to primary and high schools. The government also updated defilement laws to punish men who impregnate young girls. The law defines sexual intercourse with teens as sexual violence against young people and assigns severe punishment (Republic of Rwanda, 2012). Nevertheless, the number of teenage pregnancy cases have increased year after year (Table 1). We believe that the problem persisted because there was a dearth of comprehensive knowledge of familial and community factors associated with teenage pregnancy in Rwanda since the available data are mostly silent on contextual factors associated with it. Existing studies tend to focus on presenting the magnitude of teenage pregnancies with little emphasis on the associated factors, the examples being the surveys conducted by the National Institute of Statistics of Rwanda (NISR, 2015) and the "Collectif des Ligues et Associations de Défense des Droits de l’Homme au Rwanda" (CLADHO, 2016). NISR reports and other similar surveys did not go beyond the presentation of frequencies and percentages to indicate the magnitude of the problem. Such information can hardly provide the required knowledge to policy and decision-makers to design adequate policy measures to address the problem.

The present study intends to bridge the gap by analysing the association between household characteristics and teenage pregnancies in Rwanda. The study examines what in the household may be in association with the occurrence of pregnancy among young girls. Such knowledge potentially helps in drawing adequate preventive and intervention measures to address the problem of teenage pregnancy in Rwanda, as well as in the societies with similar social and economic status. An analysis of contextual household factors is essential for decision-makers, parents, and other stakeholders, to appropriately address the challenge with sustainable measures.

## 2 Methodology

### 2.1 Data source

Data for this study came from the Fifth Rwanda Demographic and Health Survey (RDHS-V), the largest and most comprehensive household survey conducted in Rwanda by the National Institute of Statistics of Rwanda (NISR). The NISR team collected the data for RDHS-V from November 9, 2014, to April 8, 2015, across the country using the household, and individual (women and men) questionnaires. The household questionnaire was used to collect information on the socio-demographic characteristics of household members and housing characteristics. The information was then used to identify members of households who were eligible for individual interviews (men and women). Eligible respondents were then interviewed using an individual questionnaire. The individual women questionnaire targeted women aged 15–49. The questions were about reproduction, contraception use, pregnancy, and related care, nutrition, breastfeeding, immunization, marriage, and sexual activity, fertility preferences, characteristics of a spouse, employment, adult and maternal mortality, HIV/AIDS and other sexually transmitted diseases, among other health issues.

### 2.2 Sample selection

RDHS-V got its sample through a stratified sample selected (urban and rural) from 2012 population and housing census. In each stratum, NISR team made a list of enumeration areas covering the entire stratum and therefore the entire country. A total of 492 enumeration areas were selected, comprising 113 in urban areas and 379 in rural areas. In each enumeration area, twenty-six households were randomly selected to make the sample size of 12,792 households countrywide. On the field, enumerators found one of the house units made of two households, which made a sample of 12,793. Among 12,793 households selected, 12,699 completed household questionnaire, and 13,497 women aged 15–49 completed the interviews including 2,779 teen girls aged 15–19 Countrywide.

We separated the girls' dataset from the entire women microdata files, and we merged it with the household microdata files to create one dataset for analysis purposes. The new dataset includes information on teen girls and their households. The resultant single dataset included 2779 households which contained household information and teenage girls' information. The complex nature of the sampling structure was adjusted using weighting factors added to the data files to ensure the actual representation of the results at the national level. The sampling weights were required due to the non-proportional allocation of the sample to the different districts and provinces, and possible differences in response rates.

### 2.3 Analytical framework and variable measurements

**Figure 1:**
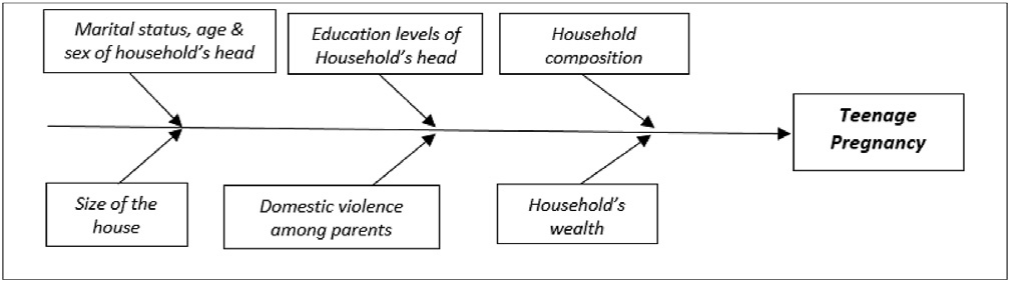
Household related factors associated with teenage pregnancy. Source: Adapted from Kirby and Lepore (2007).

We adapted the conceptual framework suggested by Kirby and Lepore (2007) and remained with the household socioeconomic factors, and referred to the previous studies to select from the database the variables that guided the framing of our analytical framework (Figure 1). The variables we selected were found significantly associated with teenage pregnancies in earlier studies conducted in developing countries similar to Rwanda (Ayele et al., 2018; Odimegwu and Mkwananzi, 2016).

The study considered ‘*teenage pregnancy*’ as an outcome variable defined as a case for a woman aged 15–19 who had had at least a live birth, ever aborted or stillbirth, or who were pregnant at the time of the survey. The study assessed teenage pregnancy as a dichotomous variable with pregnancy coded as 1, and 0 for no pregnancy.

The predictor variables were social, economic, and demographic characteristics of the head of the household. We borrowed the operationalization of the variables from the National Institute of Statistics of Rwanda. The variables include the following:

(i) *Sex of the household head,* defined as whether the household head is a male or female.
(ii) *The highest education level attained by the household head:* The variable was measured at the ordinal scale with three categories to capture the different grades completed by the household head. The three categories were the following: (i) primary level education and below, (ii) secondary and (iii) higher education level.
(iii) *Current marital status of the parents:* This is the marital status of the parents of the teen girl, defined in four categories: (i) Single (ii) Married or living together (iii) Widowed (iv) Divorced or separated.
(iv) *Age of the household head:* The dataset presented raw numbers of the age of the household head which we classified in 5 categories. The first category includes the heads of the households who were below thirty years old, and the other categories followed, with an interval of 10 years.
(v) *Size of the household:* This is the total number of individuals listed in the household roster by the household head during the survey interview. For analysis, we managed the variable into four categories as small (<5), medium (5&6), medium-large (7–9) and large families (10+). We referred to the trend of the current household size in Rwanda to divide and assign names to the categories (NISR, 2015).
(vi) *The number of bedrooms* available to the household. The dataset initially presented the number of bedrooms, and we later categorized them as 1–2; 3–5 and 6 + for analysis.
(vii) *The wealth index*: The data captured wealth index as a composite measure of the household living standard. It was calculated using easy-to-collect data on household ownership of selected assets: “television, car, mobile telephone, bicycles”; materials used for housing construction and other housing characteristics: “access to electricity, type of flooring material, number of rooms used for sleeping, and type of cooking fuel”. The scale then classified the households into the following five wealth quintiles of "*the poorest, poorer, middle, richer, and richest.*"
(viii) *Bank account ownership* depicted the status of the household in having access to money. The question was: “*does anyone in your household has a bank account*?" In case anyone has a bank account, the answer was "yes" and "no" otherwise.

### 2.4 Data analysis

We first presented the distribution of teenage pregnancies based on the household characteristics measured based on the demographic and socioeconomic characteristics of the head of the household. For the analysis of the association between household characteristics and teenage pregnancies, the study used logistic regression models of simple and multiple binary logistic regression models. The outcome variable was binary (a teen who was pregnant, or not pregnant), while all predicting variables were categorical.

## 3 Results

### 3.1 Prevalence of teenage pregnancy in Rwanda

The data in Table 2 presents the distribution of teenage pregnancies in Rwanda as of 2015, based on household socioeconomic characteristics. According to the data, male-headed households reported nearly a double of teenage pregnancy cases (64%) compared to female-headed households (nearly 36%). The data further show fluctuation in the frequency of teenage fertility according to the level of education for the head of the household. Parents with the primary school level of education and below experienced the highest rate of teenage pregnancy (85%), and the rate drops, as the parents' education level increases, to 13% and almost 2% with parents whose education level was secondary and higher respectively.

**Table 2:** Distribution of teenage pregnancy based on household characteristics.

Characteristics of the household	Status of teenage pregnancy			
	No		Yes	
	Count	%	Count	%
**Overall**	**2,578**	**92.77**	**201**	**7.23**

**Sex of the head of the household**				
Male	1,692	65.6	129	64.2
Female	886	34.4	72	35.8

**Age of the head of the household**				
Less than 30 years	143	5.5	72	35.8
30-44	840	32.6	41	20.4
45-54	874	33.9	44	21.9
55-64	473	18.3	35	17.4
65+	248	9.6	9	4.5

**Marital status of the head of the household**				
Married and living together	1,855	71.9	144	71.6
Single	84	3.3	10	5.0
Widowed	515	20.0	31	15.4
Divorced and separated	123	4.8	16	8.0

**The education level of the household head**				
Primary school and below	2,132	82.7	171	85.1
Secondary	269	10.4	27	13.4
Higher	177	6.9	3	1.5

**Size of the household**				
Small (<5)	682	26.4	96	47.8
Medium (5&6)	874	33.9	47	23.4
Medium-large (7–9)	883	34.2	46	22.9
Large (10+)	139	5.4	12	6.0

**Number of bedrooms**				
1 to 2 bedrooms	1,134	44.0	133	66.2
3 to 4	1,331	51.6	60	29.8
5 + bedrooms	113	4.4	8	4.0

**Wealth index**				
Poorest	384	14.9	45	22.4
Poor	448	17.4	40	19.9
Middle	440	17.1	38	18.9
Richer	534	20.7	32	15.9
Richest	772	29.9	46	22.9

**A bank account in the household**				
No	1,030	39.9	121	60.2
Yes	1,548	60.1	80	39.8

Regarding the age of household head, the data shows the highest proportion (36%) among households headed by individuals aged less than 30 years old, and the proportion goes down to 20% and almost 5% among households headed by individuals aged 30 to 44, and 65 + year old respectively. Based on the size of the households, the number of pregnancies varies between 48% to 23% among households with less than five members, and 5 to 6 members respectively, and drops to almost 6% among those with ten and above members.

Table 2 further presents that the frequency of teenage pregnancy cases declines as the number of bedrooms for a household increases. Among households with 1–2 bedrooms the cases were 66%, among those with 3–4 bedrooms the cases were almost 30% while they were 4% among the households with 5 + bedrooms. Additionally, the data shows the fluctuation of the number of teenage pregnancies as the household wealth index increases. Among the poorest households, the percentage was 22%, and almost 23% among the richest households, while among the poor it was 20% and 19% among the middle wealth, and 16% among the richer. Similarly, households which reported not to have a bank account had nearly a double of the teenage pregnancies (60%) compared to the households which had a bank account (40%).

The distribution of teenage pregnancies in Rwanda presents a kind of pattern when compared with the of households social and economic characteristics. We, therefore, went further to analyse the associations using logistics regression models.

### 3.2 Results of the logistic regression analysis of the association between household characteristics and teenage pregnancies in Rwanda

The results in Table 3 indicate no significant association between the sex of the household head and the occurrence of teenage pregnancy (OR: 0.91, 95% CI: 0.56–1.49). However, the age of the household head is a significant determinant of the occurrence of teenage pregnancy. The data show that families with younger household heads exhibit increased risk of teenage pregnancy, while teen girls living in households headed by individuals aged 30 to 44 are more than 90% less likely to get pregnant (OR: 0.08 with 95% CI: 0.05–0.14) compared to those living in households headed by individuals aged less than 30 years old. The trend of associations remains consistently significant as the age of the household head increases, either for the simple and the multivariate models.

**Table 3:** Results of the logistic regression analysis of the association between teenage pregnancy and household characteristics.

Variables	Simple model			Multivariate model		
	Odds Ratios	95% Confidence Interval (CI)		Odds Ratios	95% Confidence Interval (CI)	
		Lower limit	Upper limit		Lower limit	Upper limit
**Sex of the head of the household**						
Male	1.00			1.00		
Female	1.07	0.79	1.44	0.91	0.56	1.50

**Age of household head**						
Less than 30	1.00			1.00		
30-44	0.10∗	0.06	0.15	0.08∗	0.05	0.14
45-54	0.10∗	0.07	0.15	0.07∗	0.04	0.13
55-64	0.15∗	0.09	0.23	0.11∗	0.06	0.19
65+	0.07∗	0.03	0.15	0.05∗	0.02	0.11

**Marital status of household head**						
Married or living together	1.00			1.00		
Single mother	1.53	0.78	3.02	0.25∗	0.11	0.56
Widowed	0.77	0.52	1.16	1.18∗	1.06	2.04
Divorced and Separated	1.68	0.97	2.90	1.72∗	1.29	3.57

**The education level of the household head**						
Primary school and below	1.00			1.00		
Secondary	1.25	0.82	1.91	0.92	0.54	1.58
Higher	0.21∗	0.07	0.67	0.28∗	0.08	0.96

**Size of the household**						
Small (<5)	1.00			1.00		
Medium (5&6)	0.38∗	0.27	0.55	0.68∗	0.45	0.95
Medium large (7–9)	0.37∗	0.26	0.53	0.98	0.60	1.63
Large (10+)	0.61	0.33	1.14	2.13∗	1.99	4.57

**Number of bedrooms**						
1-2	1.00			1.00		
3-4	0.38∗	0.28	0.53	0.68∗	0.47	0.99
5+	0.60	0.29	1.26	1.11	0.48	2.60

**Wealth Index**						
Poorest	1.00			1.00		
Poorer	0.76	0.49	1.19	0.84	0.52	1.37
Middle	0.74	0.47	1.16	0.95	0.57	1.58
Richer	0.51∗	0.32	0.82	0.71	0.41	1.23
Richest	0.51∗	0.33	0.78	0.83	0.47	1.46

**At least one member own a bank account**						
No	1.00			1.00		
Yes	0.44∗	0.33	0.59	0.57∗	0.39	0.84

**Note:** ∗*The logistic regression analysis indicated a significant association at 95% level*.

Marital status for the household is another essential factor for teenage pregnancy in Rwanda, as the results indicate. Households headed by divorced or separated couples were 1.72 times more likely to experience teenage pregnancy compared to households headed by married couples or living together (95% CI:1.29–3.57). Similarly, households headed by widows were 1.18 times more likely to experience teenage pregnancy compared to households headed by married or living together couples (95% CI: 1.06–2.04). However, the data indicates that households headed by single mothers were less likely to experience teenage pregnancy compared to married or living together couples (OR: 0.25 with 95% CI: 0.11–0.56) (see Table 3).

Furthermore, the results indicate that the size of the household plays a significant role in teenage pregnancy in Rwanda. The results show a significant reduction of teenage pregnancies as the size of the household increases. Medium-size households were more than 60% (OR: 0.38, with 95% CI 0.27–0.55) less likely to have a teenage girl pregnant compared to small households of less than five members. The association remains consistently significant with the multivariate model (OR: 0.68, with 95% CI: 0.45–0.95). However, large families made of more than ten members were more than two times likely to experience teenage pregnancy compared to small households (OR: 2.13, with 95% CI: 1.99–4.57) in the multivariate model, while with the simple model the association was not statistically significant. The shift indicates that contextual factors around households have a significant influence on the association. Additionally, the results show that the number of bedrooms for a household is associated with teenage pregnancy where households with 3–4 bedrooms were nearly 30% less likely to experience teenage pregnancy compared to households with 1–2 bedrooms (OR: 0.68, with 95% CI: 0.47–0.99). Also, the results indicate a statistically significant association between teenage pregnancy and household possession of a bank account. Teens living in households where at least one adult member has a bank account was nearly 50% less likely to become pregnant (OR: 0.57 with 95% CI: 0.39–0.84). However, there was no statistically significant association between wealth index and teenage pregnancy.

## 4 Discussion and conclusions

This study aims to determine the household-related factors associated with teenage pregnancy in Rwanda in order to inform policy and decision-makers on the designing of measures to address it. The study revealed a significant association between teenage pregnancy and the socioeconomic status of household heads defined by age, marital status, and education level of the head of the household, and size of the household. However, the study indicated that teenage pregnancy can happen irrespective of the wealth index of the household. The number of cases among households with the lowest wealth index was closely related to that of the highest wealth index which implies that the two categories are at high risk. Although we cannot be certain of the determinants of the high risk for the two categories, we suggest, based on earlier studies, that girls from the wealthier families may be exposed to sexual risky information that comes with their access to digital devices (Mollborn, 2017; Odejimi et al., 2016), while the vulnerability for the poorest may be related to sexually-linked financial inducements to supply what parents cannot (Okigbo and Speizer, 2015). On the other hand, a household with at least one adult member that has a bank account appeared protective against teenage pregnancy. Parental use of a bank account may be a proxy of wealth or a better job, both of which may reduce teen girls' risk of sugar-daddy predation.

Further, the study indicated that the sex of the household head is not significantly associated with teenage pregnancy in Rwanda. However, there is a tendency that households led by females experience fewer cases of teenage pregnancy than when led by males. The findings allude the importance of mothers' guidance in preventing teenage pregnancy. Stokols (1996) discussed that the social environment influences behaviours; mothers create an environment of care parenting and an ambience of trust with their teen girls (Erdmans and Black, 2015) which can contribute to fewer risky partners. In this study, the results indicated a positive association between teen girls being raised by single mothers and widows and getting an adolescent age pregnancy. Besides, we observed that teen girls who stay with their both parents are less likely to get pregnant compared to those whose parents are divorced or separated.

As such, the study affirms the role of parental support in preventing unwanted pregnancies among teen girls. In a study conducted in the Philippines, Gipson & Hicks (2018) argued that a close relationship with mothers reduces risks to unwanted pregnancies among young girls. When parents separate, children are more likely to initiate sex at an early age than when the parents stay together (Kirby et al., 2007). Consistently, research conducted in Uganda found that adolescent pregnancy is significantly associated with the marital status of parents with teens living with their parents being less likely to become pregnant (Moses, 2009). In the same way, we argue that the family structure is a significant factor for unwanted pregnancies among adolescents.

The study further argues that low education levels of household heads is a significant predictor for the occurrence of teenage pregnancy. Our findings show that teens in families where the household head has a secondary and above level of education are significantly less likely to become pregnant. Odimegwu and Mkwananzi (2016) among other researchers discussed similar findings and concluded that the risk of teenage pregnancy is slightly high among adolescents whose parents have lower formal education (Kirby and Lepore, 2007; Nwogwugwu, 2013). We argue that this association relates to poor access to reproductive health knowledge and a more traditionalist orientation for parents with less education, combined with a lack of familiarity with, and confidence in contemporary methods of birth control that commonly associate with higher education levels. These tendencies amalgamate variously to diminish the content and impact of parental advice given to teen offspring regarding avoidance strategies for unwanted pregnancies.

Furthermore, the study argues that there is an association between teenage pregnancy and the size of the house, which can also be a predictor of high socioeconomic status in Rwanda (Uwizeye et al., 2020). We observed a relatively significant percentage of teenage pregnancies among households with one and two sleeping rooms. The percentage decreases considerably as the number of rooms increases. In similar studies, researchers found adverse effects on the behaviour of children raised in small and crowded houses (Foye, 2017). In a small house context, young boys and girls may prematurely be exposed to sexual acts of their parents which later induces their sexual curiosity and behaviour (Moses, 2009; Muche et al., 2017).

The high rate of teenage pregnancies among household-heads with younger age and those from low socioeconomic status were found in other studies conducted in sub-Saharan Africa as well as in other low-income context, and were associated with weak parental support and inability to satisfy the girls' financial needs (Nwogwugwu, 2013). This may lead them to engage in unsafe sex and subsequent early pregnancy. Various studies discussed that parental guidance and control over young girls are essential factors for lessening teenage pregnancy (Odejimi et al., 2016; Odimegwu and Mkwananzi, 2016; Were, 2007). Poverty and the young age of household heads put them into a weak position to advise their teen girls on risky sex (Domenico and Jones, 2007). The sexual partners of the young girls, mostly older in age, can then dupe them with money and other material gifts to the point of having unprotected sex and eventually getting pregnant (Kost et al., 2017; Nyakubega, 2008). Arguably, when household heads have the financial capacity and maturity to support teens under their household leadership, they are likely to have fewer sexual partners and less likely to be exposed to “toxic” digital information that can induce their likelihood to engage in sex and become pregnant (Domenico and Jones, 2007; UNFPA, 2013).

Generally, the study discusses the importance of household socioeconomic factors that link to teenage pregnancy in Rwanda. We conclude that financial, social and educational level of empowerment of parents, and households in which parents agree harmoniously on boundary-setting, child discipline and anti-pregnancy messaging, contribute to lowering the cases of teenage pregnancy. Social and economic support to teen girls which include parental supervision, guidance, and financial care are essential aspects to consider in order to reduce teenage pregnancy rates. The study, therefore, informs that strategies for addressing the increase of teenage pregnancy should target establishing harmony within households and empowering parents to support their daughters both mentally and financially. Equally important, interventions would aim to improve opportunities for parents irrespective of their socioeconomic status to access sexual and reproductive health education for teens, the knowledge the parents will use to support their daughters.

## 5 Strengths, limitations and suggestions for further studies

The major strength of this study lies on the use of data from a nationwide survey (DHS) which provides a comprehensive view of teenage pregnancies, and associated household factors in Rwanda. The findings are potentially generalizable to other countries with a similar level of socioeconomic status. However, the study has an essential limitation as a secondary analysis of the DHS data, originally cross-sectional, and household-based. The type of study does not permit causal-effect attribution of the observations made. Also, the surveys omit homeless and institutionalized children, which may bias our results.

Future studies may address the limitations through qualitative inquiries of the reasons young women become pregnant and decide to keep their babies despite the social and family related challenges. Also, further studies may investigate the views and roles of teen boys and men both as an essential part of the problem, and as often-overlooked stakeholders in building a solution.

## Declarations

### Author contribution statement

Dieudonne Uwizeye, Ruben Muhayiteto, Evelyne Kantarama, Yusuf Murangwa, Simeon Wiehler: Analyzed and interpreted the data; Wrote the paper.

### Funding statement

This research did not receive any specific grant from funding agencies in the public, commercial, or not-for-profit sectors.

### Competing interest statement

The authors declare no conflict of interest.

### Additional information

At the time of writing, Dieudonne Uwizeye was supported by a Postdoctoral fellowship funded through the Consortium for Advanced Research Training in Africa (CARTA). CARTA is jointly led by the African Population and Health Research Center and the University of the Witwatersrand and funded by the Carnegie Corporation of New York (Grant No--B 8606.R02), Sida (Grant No:54100113), the DELTAS Africa Initiative (Grant No: 107768/Z/15/Z) and Deutscher Akademischer Austauschdienst (DAAD). The DELTAS Africa Initiative is an independent funding scheme of the African Academy of Sciences (AAS)'s Alliance for Accelerating Excellence in Science in Africa (AESA) and supported by the New Partnership for Africa's Development Planning and Coordinating Agency (NEPAD Agency) with funding from the Wellcome Trust (UK) and the UK government. The statements made and views expressed are solely the responsibility of the Fellow and his co-authors.
